# Experimental Infection of the Pig with *Mycobacterium ulcerans*: A Novel Model for Studying the Pathogenesis of Buruli Ulcer Disease

**DOI:** 10.1371/journal.pntd.0002968

**Published:** 2014-07-10

**Authors:** Miriam Bolz, Nicolas Ruggli, Marie-Thérèse Ruf, Meret E. Ricklin, Gert Zimmer, Gerd Pluschke

**Affiliations:** 1 Swiss Tropical and Public Health Institute, Basel, Switzerland; 2 University of Basel, Basel, Switzerland; 3 Institute of Virology and Immunology (IVI), Mittelhäusern, Switzerland; University of Tennessee, United States of America

## Abstract

**Background:**

Buruli ulcer (BU) is a slowly progressing, necrotising disease of the skin caused by infection with *Mycobacterium ulcerans*. Non-ulcerative manifestations are nodules, plaques and oedema, which may progress to ulceration of large parts of the skin. Histopathologically, BU is characterized by coagulative necrosis, fat cell ghosts, epidermal hyperplasia, clusters of extracellular acid fast bacilli (AFB) in the subcutaneous tissue and lack of major inflammatory infiltration. The mode of transmission of BU is not clear and there is only limited information on the early pathogenesis of the disease available.

**Methodology/Principal Findings:**

For evaluating the potential of the pig as experimental infection model for BU, we infected pigs subcutaneously with different doses of *M. ulcerans*. The infected skin sites were excised 2.5 or 6.5 weeks after infection and processed for histopathological analysis. With doses of 2×10^7^ and 2×10^6^ colony forming units (CFU) we observed the development of nodular lesions that subsequently progressed to ulcerative or plaque-like lesions. At lower inoculation doses signs of infection found after 2.5 weeks had spontaneously resolved at 6.5 weeks. The observed macroscopic and histopathological changes closely resembled those found in *M. ulcerans* disease in humans.

**Conclusion/Significance:**

Our results demonstrate that the pig can be infected with *M. ulcerans*. Productive infection leads to the development of lesions that closely resemble human BU lesions. The pig infection model therefore has great potential for studying the early pathogenesis of BU and for the development of new therapeutic and prophylactic interventions.

## Author Summary

Buruli ulcer caused by *Mycobacterium ulcerans* infection is a necrotizing disease of the skin and the underlying subcutaneous tissue. Since the skin of pigs (*Sus scrofa*) has striking structural and physiological similarities with human skin, we investigated whether it is possible to develop an experimental *M. ulcerans* infection model by subcutaneous injection of the mycobacteria into pig skin. Injection of 2×10^6^ or 2×10^7^ colony forming units of *M. ulcerans* led to the development of lesions that were both macroscopically and microscopically very similar to human Buruli ulcer lesions. In particular for the characterization of the pathogenesis of Buruli ulcer and of immune defence mechanisms against *M. ulcerans*, the pig model appears to be superior to the mouse foot pad model commonly used for the evaluation of the efficacy of chemotherapeutic regimens.

## Introduction

Buruli ulcer (BU), caused by infection with *Mycobacterium ulcerans*, is a human disease of the skin primarily affecting subcutaneous fat tissue and leading to ulceration of the overlying dermal and epidermal layers [1], [2]. The disease is reported from countries worldwide but has its highest prevalence in West Africa [3]. Natural reservoirs of *M. ulcerans* as well as the mode(s) of transmission are not clearly identified [3], [4]. While for a long time wide surgical excision was the only treatment option for BU, since 2004 the World Health Organization (WHO) recommends antibiotic therapy with rifampicin and streptomycin for 8 weeks [5]. This change in standard treatment has reduced recurrence rates to less than 2% [6]–[9].

*M. ulcerans* produces the polyketide exotoxin mycolactone that is responsible for the necrotizing nature of BU [10]. Three distinct non-ulcerative stages of the disease are described: subcutaneous, painless and movable nodules or papules, oedema and plaques. All three stages may progress to ulceration once the destruction of the subcutis results in collapse of the overlying epidermis and dermis [11].

Ulcerative BU lesions have been histopathologically well described through the analysis of excised tissue from surgically treated patients. Coagulative necrosis, fat cell ghosts and epidermal hyperplasia together with the presence of extracellular clusters of acid fast bacilli (AFB) in the absence of major inflammatory infiltrates in central parts of the lesions are considered hallmarks of the disease and can also be used for histopathological diagnosis [12], [13]. However, early, pre-ulcerative stages have been described less frequently, because in particular in the African BU endemic regions patients are rarely reporting at treatment centres during early stages of the disease. Furthermore, with the replacement of surgical treatment by chemotherapy, tissue samples are not easily available any longer. Therefore, a suitable experimental animal infection model is required to contribute to the understanding of early host-pathogen interactions and pathogenesis in BU.

A range of animal species have been reported of being naturally infected with *M. ulcerans* and of developing ulcerative lesions. These include koalas, possums, cats, dogs and horses [14]–[21]. Except for possums which appear to be unusually susceptible to the disease, these animal infections seem to occur only sporadically [22]. Experimental *M. ulcerans* infections have been performed with amphibians, armadillos, rats, mice, guinea pigs and monkeys, with a mouse foot pad model being most widely used for studying the efficacy of prophylactic and therapeutic interventions [23]–[29]. Here we propose the pig (*Sus scrofa*) as experimental *M. ulcerans* infection model, since pigs are closely related to humans in terms of many aspects of anatomy and physiology [30], [31]. The pig is widely used as a model in dermatological studies because pig skin, in contrast to rodent skin, has striking similarities to human skin [32]. Not only the thickness of the epidermis and the dermis are comparable to human skin [33], but also the presence of a subcutaneous fat cell layer is favouring the pig model over the mouse foot pad model commonly used for analysing BU pathogenesis. Furthermore, the porcine immune system reflects the human immune system in many aspects better than the murine immune system does [34], [35]. For all these reasons we explored here the potential of the pig to serve as model for human *M. ulcerans* infection.

## Materials and Methods

### Ethical statement

All animal experiments described here were approved by the Animal Welfare Committee of the Canton of Berne under licence number BE50/11, and conducted in compliance with the Swiss animal protection law and with other national and international guidelines.

### Bacteria

The *M. ulcerans* strain used in this study was isolated in 2010 from a swab taken from the undermined edges of the ulcerative lesion of a Cameroonian BU patient [4]. Five passages of the strain after isolation were done in Bac/T medium (Biomerieux) at 30°C. For preparation of the inoculum, bacteria were cultivated in Bac/T medium for 6 weeks, recovered by centrifugation and diluted in sterile phosphate-buffered saline (PBS) to 375 mg/ml wet weight corresponding to 2×10^8^ CFU/ml as determined by plating serial dilutions on 7H9 agar plates. From this stock solution suspension serial dilutions in PBS were prepared for infection.

### Infection and inoculation with synthetic mycolactone A/B

Specific pathogen-free 2-month-old pigs (Large White) from the in-house breeding unit of the Institute of Virology and Immunology (IVI) were kept under BSL3 conditions one week prior and during the time of experimental infection. Animals were checked once daily for macroscopic signs of infection, had *ad libitum* access to water and were fed daily with complete pelleted food.

Pigs were infected on both flanks at four to six infection sites with 100 µl of *M. ulcerans* suspension, containing 2×10^7^, 2×10^6^, 2×10^5^, 2×10^4^ or 2×10^3^ CFU. Injection areas were wiped with 70% ethanol and bacterial suspensions injected subcutaneously with a 26G needle. Individual infection sites were encircled with a black marker and the labelling renewed at least once a week. Animals were euthanized at 2.5 weeks or 6.5 weeks post-infection and tissue samples taken as described below.

In addition, the effect of mycolactone was studied directly by injecting 5 µg or 0.5 µg of synthetic mycolactone A/B [36] and analysing tissue specimens taken 2.5 weeks later.

### Euthanasia and necropsy

Pigs were euthanized by intravenous injection of pentobarbital (150 mg/kg bodyweight) and subsequent exsanguination. Skin tissue at infection sites was extensively excised with a scalpel and scissors, including all layers of the skin down to the fascia, and samples were immediately transferred to 10% neutral-buffered Formalin solution (approx. 4% formaldehyde).

### Histopathological analysis

After fixation samples were transferred to 70% ethanol for storage and transport, dehydrated and embedded into paraffin. 5 µm thin sections were cut, deparaffinised, rehydrated and directly stained with Haematoxylin/Eosin (HE) or Ziehl-Neelsen/Methylene blue (ZN) according to WHO standard protocols [11]. Stained sections were mounted with Eukitt mounting medium (Fluka). Pictures were taken with a Leica DM2500B microscope or with an Aperio scanner.

## Results

### Macroscopic appearance of *Mycobacterium ulcerans* infected pig skin

**Figure 1:**
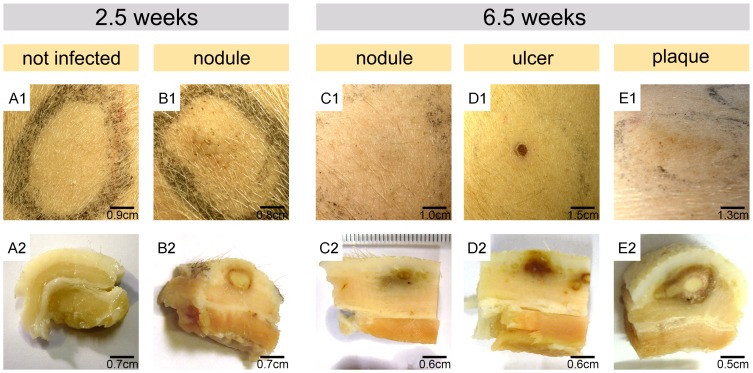
Macroscopic appearance of pig skin infected with *M. ulcerans*. Development of representative lesions 2.5 weeks or 6.5 weeks after subcutaneous infection with 2×10^7^ (B1, D1 and E1) or 2×10^6^ (C1) CFU is depicted and compared to a site left uninfected (A1). Excised tissue specimens were fixed and vertically cut in half to visualize macroscopically visible alteration in tissue structure (A2–E2). For high inoculation doses (≥2×10^6^ CFU) the formation of nodules (B1) with necrotic centres was observed already 2.5 weeks after infection (B2). These yellow centres indicative for coagulative necrosis were surrounded by a reddish ring (B2). At 6.5 weeks after infection, these nodules had progressed into a small ulcer or a plaque (D1, E1) associated with marked macroscopically visible alterations in tissue structure (D2, E2). At sites injected with 2×10^6^ CFU nodules with greyish discoloration of the dermis had developed 6.5 weeks after injection of *M. ulcerans* (C1, C2).

In order to assess early effects of the subcutaneous experimental infection of pigs with doses of 2×10^3^ to 2×10^7^ *M. ulcerans* CFU, injection sites were closely monitored for macroscopic changes of the skin. At 2.5 weeks after injection of the bacteria, first changes in colouration and thickness of the skin became apparent at the sites inoculated with the highest inoculation doses, 2×10^7^ and 2×10^6^ CFU (Fig. 1, B1). Like nodular BU lesions in humans, these early lesions were elevated, movable, firm and palpable. When these skin areas were excised 2.5 weeks and 6.5 weeks after infection and vertically cut in half after fixation in formalin, roundish yellow structures reflecting coagulative necrosis in the dermis became macroscopically apparent (Fig. 1, B2). A belt with reddish colour, reflecting infiltrating cells and bleeding into the skin, was observed around the necrotic core. While these structures were larger at sites inoculated with a dose of 2×10^7^ CFU than at sites inoculated with 2×10^6^ CFU, the general architecture observed with both inoculation doses was similar. At sites inoculated with <2×10^5^ CFU, no macroscopically visible alterations of the skin were found 2.5 weeks after infection (Fig. 1, A1 and A2).

At 6.5 weeks after experimental infection, sites injected with the highest inoculation dose had either enlarged to an indurated plaque (Fig. 1 E1) or ulcerated (Fig. 1, D1). At sites injected with 2×10^6^ CFU, nodular lesions were observed that were flatter and less palpable compared to those detected 2.5 weeks after infection (Fig. 1, C1). These lesions were macroscopically clearly visible in cross sections through the tissue (Fig. 1, C2). Nodular and ulcerative lesions exhibited greyish/reddish colour changes in the dermis and subcutis (Fig. 1, C2 and D2). The plaque lesion developing after injection with 2×10^7^ CFU appeared as long cord-like structure with a centre made of yellowish necrotic slough, surrounded by several layers differing in colouration (Fig. 1, E2).

### Histopathological features of the pig skin 2.5 weeks after experimental infection

**Figure 2:**
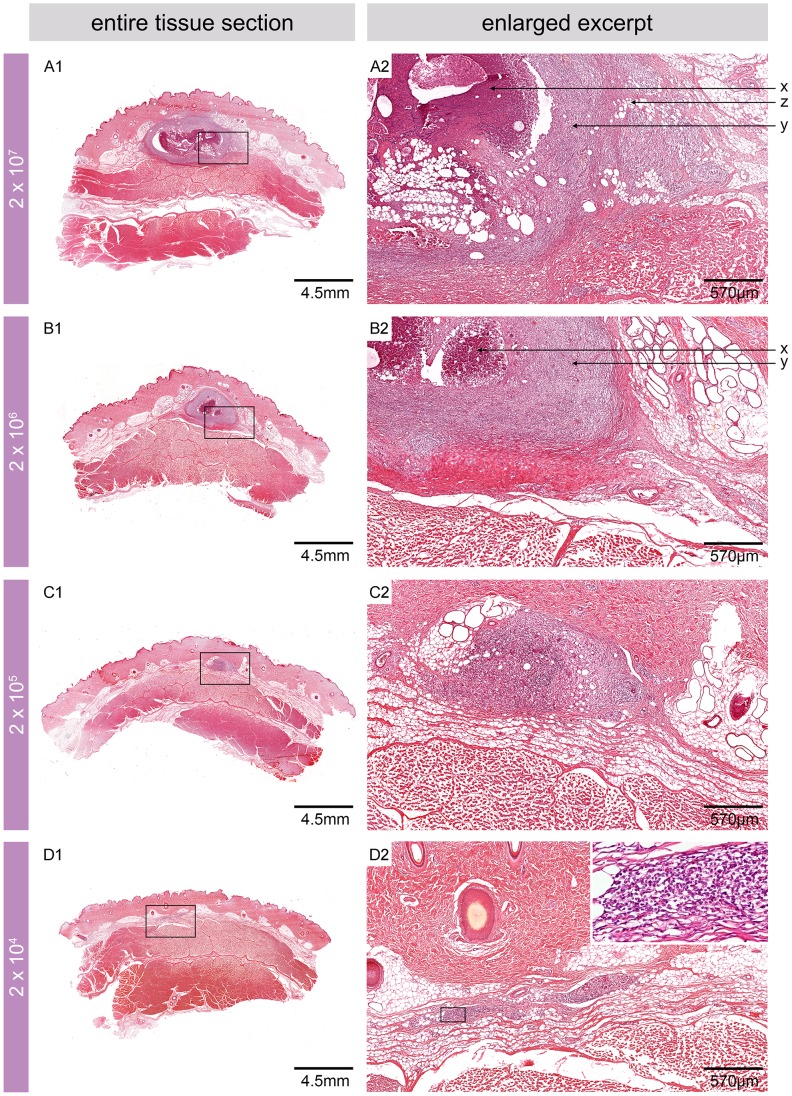
Microscopic appearance of pig skin 2.5 weeks after experimental infection. Histologic sections stained with HE. Infiltrating cells were found in the fat layer between dermis and muscle tissue at sites infected with ≥2×10^4^ CFU (A1, B1, C1 and D1). While the two highest inoculation doses led to the development of lesions with a necrotic core surrounded by strong infiltration (A2, B2), infiltration but no necrotic core was observed when doses of 2×10^4^ and 2×10^3^ CFU were used (C2, D2 insert). Fat cell ghosts were found at sites infected with the three highest inoculation doses (A2, B2 and C2). x: necrosis, y: infiltration, z: fat cell ghosts.

Microcopically, infiltrating immune cells were found 2.5 weeks after infection at all sites inoculated with ≥2×10^4^ CFU (Fig. 2, A1, B1, C1 and D1). As expected from the macroscopically observed signs, the most pronounced histopathological alterations were associated with the two highest inoculation doses (2×10^7^ and 2×10^6^ CFU). The non-ulcerative lesions that developed between the dermis and the underlying muscle tissue displaced the fat layer (Fig. 2, A1 and B1) and caused the macroscopically visible elevation of the skin (Fig. 1, B1). Microscopically, a necrotic core surrounded by large numbers of infiltrating cells and interspersed with fat cell ghosts was observed (Fig. 2, A2 and B2).

At sites infected with 2×10^5^ CFU, no necrotic core structures but some fat cell ghosts and accumulations of infiltrating cells were found (Fig. 2, C1 and C2). The infection with 2×10^4^ CFU caused a small accumulation of infiltrating cells (Fig. 2, D1 and D2) and no signs of infection and/or inflammation were observed at sites inoculated with the lowest dose (2×10^3^ CFU).

### Histopathological features of the pig skin 6.5 weeks after experimental infection

**Figure 3:**
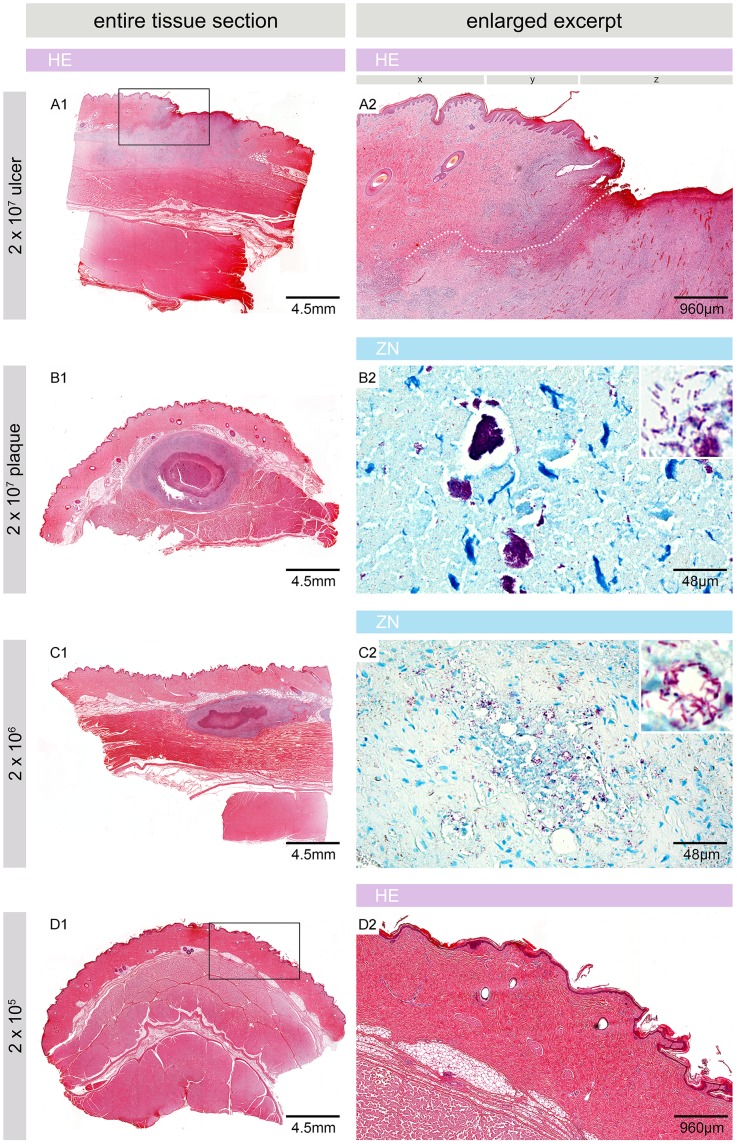
Microscopic appearance of pig skin 6.5 weeks after experimental infection. Histologic sections stained with Haematoxylin/Eosin (HE) (A1, A2, B1, C1, D1 and D2) or Ziehl-Neelsen/Methylene blue (ZN) (B2, C2). At sites injected with 2×10^7^ CFU nodules had either developed into a small ulcer with destroyed epidermis (z), strong infiltration and indications for the development of undermined edges (A1, A2, dotted line) or into a plaque with a necrotic core surrounded by infiltrating cells (B1). x: intact epidermis, y: epidermal hyperplasia, z: destroyed/missing epidermis. The site infected with 2×10^6^ CFU showed a similar architecture as the plaque but flatter, less organized and with a smaller overall circumference (C1). Both lesions comprised AFB in their necrotic cores, either in big clumps (B2) or in smaller numbers and smaller aggregations (C2). No signs of infection, inflammation and pathology were observed at sites inoculated with 2×10^5^ CFU (D1, D2) or less (not shown).

At 6.5 weeks after experimental infection, histopathological changes were only found at sites that had been injected with 2×10^7^ or 2×10^6^ CFU. In contrast, the skin appeared macro- and microscopically healthy following infection with lower doses of *M. ulcerans*, exhibiting intact epidermis and fat cells, undistorted collagen fibre networks and no marked inflammatory infiltration (Fig. 3, D1 and D2).

**Figure 4:**
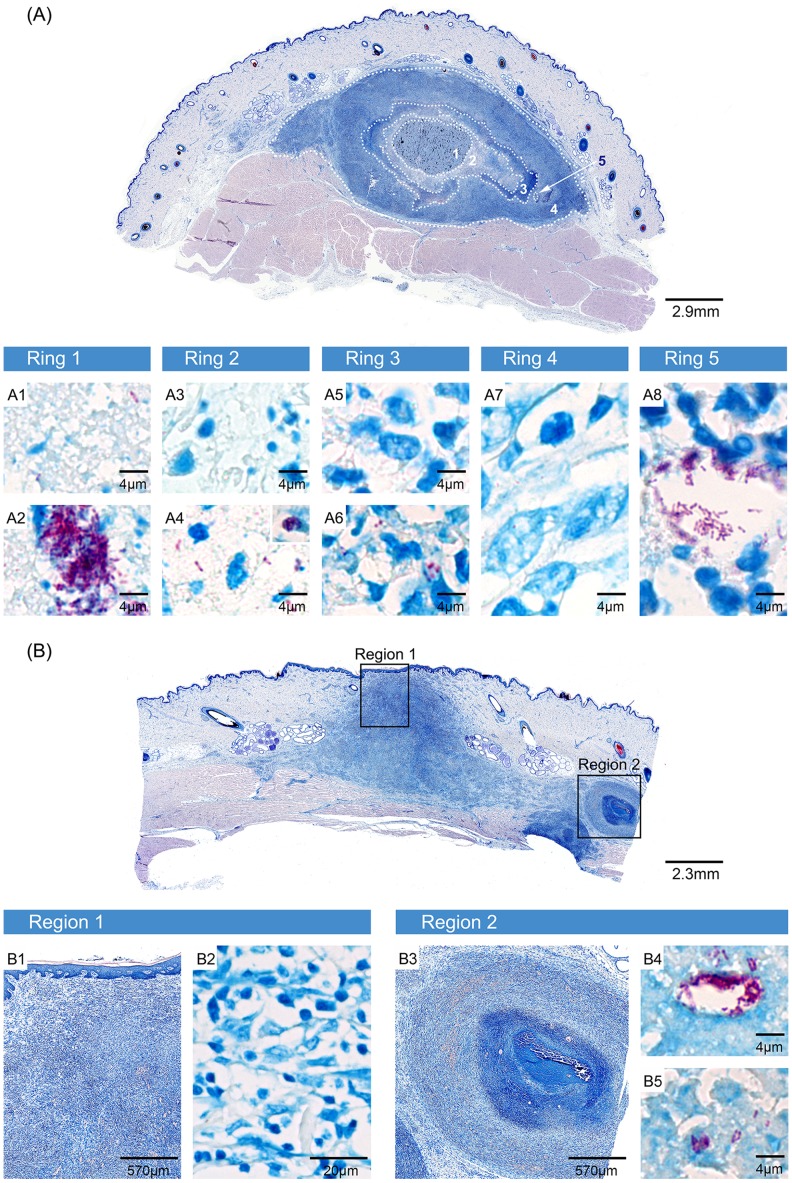
Containment of large amounts of AFB in the necrotic core and development of satellite microcolonies. Histologic sections stained with ZN. A plaque (A) and a small ulcer (B) are shown that developed 6.5 weeks after infection with 2×10^7^ CFU. The ulcerated lesion was strongly infiltrated at the site of ulceration, where no AFB were found (Region 1, B1, B2). Lateral and between dermis and muscle tissue infiltrating cells enclosed small necrotic areas (Region 2, B3), where AFB were found as satellite microcolonies (B4, B5). The plaque consisted of distinct layers of infiltrating cells encasing a necrotic core containing large clumps of bacteria (Ring 1, A1, A2). A second and third ring with decreasing bacterial load and integrity and increasing integrity of infiltrating cells were layered around this core (Ring 2, A3, A4 and Ring 3, A5, A6). A belt of intact cells was surrounding these three inner layers. It did not contain any AFB (Ring 4, A7) except for a microcolony peripheral to the main bacterial burden (Ring 5, A8).

Where the infection focus had started to ulcerate, strong infiltration towards the destroyed epidermis was observed (Fig. 3, A1 and A2). No AFB were found in this region, indicative for loss of the necrotic core with the major burden of AFB through the ulceration (Fig. 3, A2, Fig. 4, B1 and B2). Small clusters of AFB were found at deeper sites in the tissue, lateral to the ulceration site (Fig. 4, B3–B5). Infiltration and destruction of collagen fibres extended into the lower part of the dermis and the upper part of the subcutis, reaching far beyond the area where the epidermis was destroyed (Fig. 3, A1), indicating the formation of undermined edges (Fig. 3, A2, dotted line).

The overall architecture of the plaque lesion that had developed resembled the nodular stages seen 2.5 weeks after infection, i.e. a necrotic centre was surrounded by layers of infiltrating cells (Fig. 3, B1). While large clumps of extracellular AFB were found in the necrotic core after injection of 2×10^7^ CFU (Fig. 3, B2), AFB were less abundant and bacterial clumps smaller when 2×10^6^ CFU were used for infection (Fig. 3, C2). Fig. 4A depicts the complex architecture of a plaque lesion (2×10^7^ CFU dose) with several distinct belts of infiltrating cells surrounding a central necrotic core which contained huge clusters of AFB but was completely devoid of infiltration (Fig. 4A, Ring 1, A1 and A2). In the surrounding ring 2, AFB were scarce and had mostly a beaded appearance. In addition to these single AFB, small globi-like clusters of AFB were found, along with Methylene blue stained remains of infiltrating cells (Fig. 4A, Ring 2, A3 and A4). Ring 3 contained mostly small infiltrating cells that appeared intact, and some acid-fast bacterial debris (Fig. 4A, Ring 3, A5 and A6). The outermost layer that could be distinguished did not contain AFB and was mainly built by macrophages and lymphocytes (Fig. 4A, Ring 4, A7). Hence, the number and integrity of AFB decreased from the centre to the periphery of the lesion, whereas the integrity of the cellular infiltration showed an opposite trend, most likely reflecting levels of the cytotoxic macrolide mycolactone decreasing from centre to periphery.

### Histopathological resemblance of pig and human BU lesions

**Figure 5:**
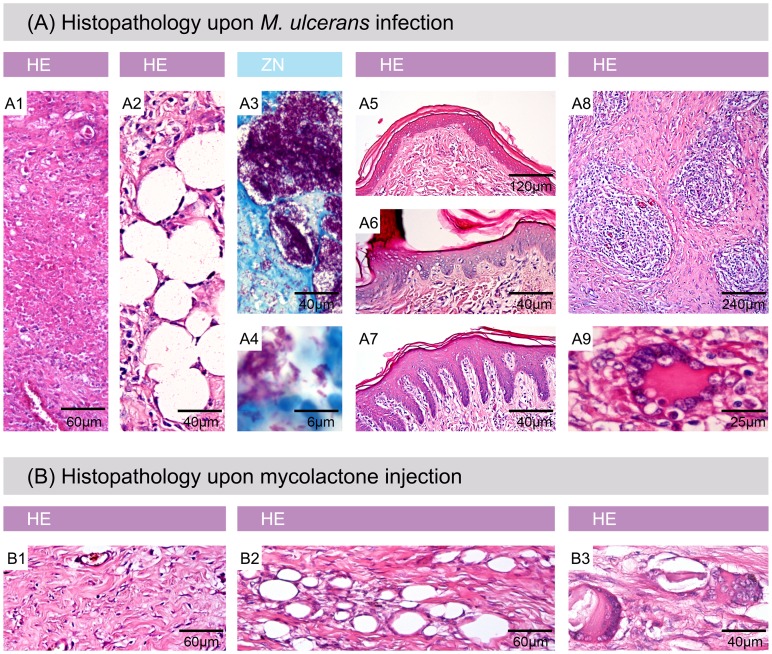
Histophathological hallmarks of Buruli ulcer in experimentally infected pig skin. Histologic sections stained with Haematoxylin/Eosin (HE) (A1, A2, A5, A6, A7, A8, A9, B1, B2 and B3) or Ziehl-Neelsen/Methylene blue (ZN) (A3, A4). A: All typical histopathological features of BU in humans were found in infected pig skin. A1: necrosis, A2: fat cell ghosts, A3 and A4: extracellular clusters of AFB, A5: healthy epidermis, A6: moderate epidermal hyperplasia, A7: strong epidermal hyperplasia, A8: granuloma formation, A9: giant cells. B: Histopathological changes induced by mycolactone injection. B1: necrosis, B2: fat cell ghosts, B3: giant cells.

All key features of BU pathology in humans were also found in the experimentally infected pig skin. Already 2.5 weeks after infection, coagulative necrosis (Fig. 5, A1), fat cell ghosts (Fig. 5, A2) and extracellular clusters of AFB (Fig. 5, A3 and A4) were detected. Slight epidermal hyperplasia was already observed at 2.5 weeks and became more pronounced 6.5 weeks after infection (Fig. 5, A5–A7). At this time, typical histopathological hallmarks of more advanced human BU lesions also emerged in the infected pig skin, namely formation of granulomas (Fig. 5, A8) and presence of giant cells (Fig. 5, A9). Not only experimental infection with *M. ulcerans* led to these typical alterations in the skin, but also the injection of synthetic mycolactone A/B (Fig. 5B).

### Development of satellite infection foci

Besides the general histopathological changes, another similarity to findings in human BU [37] was observed: the formation of satellite infection foci adjacent to the primary lesion. A striking example for this is depicted in Fig. 4B where two satellite foci with small clusters of AFB in a necrotic core were found peripheral to the ulcerated main infection focus (Fig. 4B, Region 2). Likewise in the plaque lesion depicted in Fig. 4A, clusters of AFB were found near the main infection focus (Fig. 4A, Ring 5, A8).

## Discussion

Detailed studies on the early pathogenesis of BU in an animal model closely mimicking human BU would be very important for a better understanding of host-pathogen interactions and the relative importance of different effector functions of the innate and adaptive immune system against *M. ulcerans*. Here we explored the potential of the pig to serve as model for human *M. ulcerans* infection. After having infected pigs subcutaneously with high doses (2×10^6^ or 2×10^7^ CFU) of *M. ulcerans* bacteria, we observed the development of different forms of BU lesions (nodules, plaques and ulcers). Macroscopic and histopathological changes closely mirrored human BU. Challenge with lower doses (2×10^3^ to 2×10^5^ CFU) resulted in limited tissue destruction and/or infiltration 2.5 weeks after infection, which resolved spontaneously until week 6.5. Likewise, the dose of bacteria transmitted may be of critical importance for the outcome of a natural *M. ulcerans* infection in humans. Sero-epidemiological analyses in human populations living in BU endemic areas have indicated that exposure to *M. ulcerans* often leads to self-resolving, non-symptomatic infections, as indicated by development of *M. ulcerans* specific antibody responses [38], [39]. While macrophages and other immune cells might be able to eliminate smaller numbers of scattered *M. ulcerans* cells, microcolonies of a critical size may develop a protective cloud of mycolactone around them. If the local concentration of the macrolide cytotoxin exceeds a certain level, infiltrating cells may be killed before they can reach the bacteria. This leads to the characteristic picture of clusters of extracellular AFB located primarily in the necrotic core of advanced lesions, which is devoid of living infiltrating immune cells, but contains debris of early inflammatory infiltrates [40], [41].

In our study, we observed round elevations of the skin already 2.5 weeks after infection. These alterations were firm, movable and clearly palpable and hence displayed the characteristic features of human BU nodules [11]. Microscopic investigation of the infected skin sites revealed that most histopathological hallmarks of BU had already developed during the first 2.5 weeks of infection if 2×10^6^ or 2×10^7^ CFU of *M. ulcerans* was used. The experimentally induced nodules exhibited a necrotic core containing extracellular AFB surrounded by infiltrating cells and fat cell ghosts. Subcutaneous injection of the bacteria led to the formation of an infection focus in the lower dermis and subcutis, where it is also typically found in human BU [13].

In ulcerative human BU lesions AFB are typically focally distributed and not evenly dispersed in the affected tissue [37], [42]. Ulceration leads to the shedding of necrotic tissue containing masses of AFB. Therefore the bacterial burden is usually higher in non-ulcerative lesions than in ulcers, where the majority of the remaining AFB reside in the undermined edges of the ulcers. Our histopathological analyses showed, that like in human BU disease [37], satellite lesions may develop near the primary lesion. These may emerge from globi-like accumulations of AFB originating from bacteria that were internalized and transported to distant sites by phagocytic cells. Globi-like accumulations are also found in human BU [43] and in experimentally infected mice [44]–[46]. Again, these microcolonies may have to reach a critical size to be able to develop a protective cloud of mycolactone around them. The emergence of only small numbers of newly established microcolonies may explain why borders of advanced ulcers often appear to be very heterogeneous with respect to disease activity with some regions displaying progressive tissue destruction and others showing spontaneous healing tendencies.

At 6.5 weeks after infection with 2×10^6^ or 2×10^7^ CFU of *M. ulcerans*, lesions that were still closed comprised a necrotic centre containing clumps of AFB surrounded by well stratified belts of infiltrating cells. Similarly, lesions consisting of a necrotic core surrounded by an inner belt of CD14 positive monocytes/macrophages and a more external belt of CD3 positive T-cells have been described in human BU [47]. The integrity and number of bacteria was decreasing to the outer rim of the lesion. In contrast, the density and integrity of the cellular infiltrates decreased towards the necrotic core.

In the pig model first macroscopic signs of infection (nodules) developed relatively fast after injection of a high number of bacteria. For human BU disease in Uganda and Southern Australia incubation periods of 4–13 weeks and 5–38 weeks have been estimated, respectively [48]. However, incubation periods as short as 2–3 weeks have also been described [49]. Despite extensive analyses we did not find bacteria in the tissue with low inoculation doses at the 6.5 week time point. Therefore we assume that also at later time points lesions would not develop with these low infection doses. It is possible that pigs are more resistant to *M. ulcerans* infection than humans. Consequently, the size of the inoculum to achieve productive experimental infection may be higher for pigs than for the natural infection of humans. This high experimental inoculation dose may have led to fast progression of the disease.

In conclusion, our findings indicate that the pig is a very good animal model to study many aspects of *M. ulcerans* infection. Pig skin represents a much closer model for human skin than murine foot pads, ears or tails with respect to physiology, structure and abundance of fat tissue [50]. In addition, the immune system of the pig resembles the human system more closely than that of the mouse [34]. In particular the development of new therapeutic and prophylactic interventions might benefit from the porcine *M. ulcerans* infection model.
